# Intensified-Dose Chemotherapy in Combination With Gemtuzumab-Ozogamicin for the Treatment of Favorable-Risk Acute Myeloid Leukemia (AML)

**DOI:** 10.7759/cureus.26240

**Published:** 2022-06-23

**Authors:** Rathnamitreyee Vegunta, Ronen Harel, Amir Steinberg

**Affiliations:** 1 Internal Medicine, Westchester Medical Center, Valhalla, USA; 2 Hematology and Oncology, Crystal Run Healthcare, Middletown, USA; 3 Hematology and Oncology, Westchester Medical Center, Valhalla, USA

**Keywords:** thrombocytopenia, npm1, gemtuzumab – ozogamicin, intensified daunorubicin, aml

## Abstract

Chemotherapy has been the standard of treatment for acute myeloid leukemia (AML). With the emergence of new therapies for AML like gemtuzumab-ozogamicin and FLT3 inhibitors, such as sorafenib, midostaurin, and gilteritinib, the optimal dose of chemotherapy and safety profile in different age groups when combined with these new therapies is yet to be established. There are limited data on the treatment of AML by combining intensified daunorubicin (doses of 90 mg/m^2^) with gemtuzumab-ozogamicin (GO). We report a young adult with favorable-risk AML treated with daunorubicin at a dose of 90 mg/m^2^ combined with GO, who had a complete response after induction but had a profound nadir of platelet count after induction and consolidation.

## Introduction

Chemotherapy has been the backbone of the treatment of acute myeloid leukemia (AML). Targeted treatment (FLT3 inhibitors and isocitrate dehydrogenases (IDH) inhibitors) and others (i.e. venetoclax) are now approved for AML treatment. The safe and optimal combination of these new therapeutic agents with standard chemotherapy remains a key question, particularly if intensification of the backbone chemotherapy regimen is still relevant. Some clinical trials reported the benefits of intensified daunorubicin when compared to the standard dose in combination with FLT3 inhibitors. Gemtuzumab-ozogamicin (GO), an anti-CD33 monoclonal antibody linked to a DNA-damaging cytotoxic drug, was developed after the discovery that anti-CD33 antibodies bind selectively to AML cells and are internalized across the tissue membrane. A retrospective study published by Singh A et al. demonstrated the safety of intensified daunorubicin and GO and the combination with FLT3 inhibitors in patients with favorable-risk AML [1]. The optimal chemotherapy combination with these new agents for the management of young AML patients remains unclear.

## Case presentation

A 50-year-old woman with recurrent headaches was found to have leukocytosis at an outside hospital and was transferred to our hospital. Her past medical history was significant for celiac disease, emphysema, arthritis, fibromyalgia, and chronic regional pain syndrome. Her home medications include bronchodilators and pain medications. The patient denied any history of malignancy in her family. She was tachypneic and hypoxic and required oxygen supplementation on arrival. Labs were notable for a white blood cell count of 38.2 k/mm^3^ with 72% blasts with monocytic differentiation, hemoglobin 10.2 gm/dl, platelet count 113 k/mm^3^, fibrinogen >600 mg/dL, lactate dehydrogenase 604 U/L, and uric acid 4.8 mg/dL. Bone marrow biopsy and aspiration with flow cytometry revealed 89% myeloid blasts and findings consistent with acute myeloid leukemia (AML) non-M3 phenotype. Given her acute presentation, we elected to begin induction shortly after preliminary pathology results. She was treated with cytarabine 200 mg/m^2^ and intensified daunorubicin 90 mg/m² per day for three days (7+3 regimen) for induction. The results of the next-generation sequencing (NGS) panel for AML came a week later. NPM1 was detected and other mutations, including FLT3, were negative. No other cytogenetic abnormalities were detected. Based on data supporting the use of gemtuzumab-ozogamicin (GO) in favorable-risk AML [2], we added GO for Day 7 and Day D10. Her induction course was complicated by thrombocytopenia with platelet count <50 k/mm^3^ for four weeks and reached a nadir of 1 k/mm^3^.

The platelet count was <10 k/mm^3^ for 11 days post-induction. Other complications post-induction were neutropenic fever, bacteremia with vancomycin-resistant Enterococcus, Staphylococcus epidermidis, colitis, and a rectal abscess, which required surgical drainage. The patient was in complete remission after induction and has had three cycles of consolidation thus far with high-dose cytarabine (3 g/m^2^) and GO. Pathology from bone marrow revealed complete remission with no more NPM1 or other mutations.

## Discussion

A retrospective study published by Singh A et al. demonstrated the safety of intensified daunorubicin and GO and the combination with FLT3 inhibitors in patients with favorable-risk AML [1]. A phase 3 randomized trial by Lee JH et al. compared high-dose daunorubicin (HD-DN, 90 mg/m² per day for three days) with standard-dose daunorubicin (SD-DN, 45 mg/m² per day for three days) as induction chemotherapy in addition to cytarabine (200 mg/m² per day times seven days) in adults with AML. HD-DN improved both the complete response (CR) (82.5% vs 72%; p = 0.014) and overall survival (46.8% vs 34.6%; p = 0.030) compared to SD-DN [3]. However, the ALFA-701 trial, which combined GO with induction chemotherapy and showed the benefit of combining daunorubicin and GO particularly in favorable/intermediate-risk groups, incorporated a dose of 60 mg/m^2^ of daunorubicin. This study had been recruiting patients before the Lee JH et al. paper results had been published showing the benefits of higher doses of daunorubicin [3]. Additionally, the ALFA-701 trial focused on patients ages 50-70 years old for whom higher doses of daunorubicin might not have been as well-tolerated [4].

In the retrospective study by Singh A et al., the authors reported on the outcomes of 37 AML patients treated with GO-based induction. Nine of the patients were treated with dose-intensified GO-based induction (daunorubicin dose 90 mg/m^2^ with cytarabine) and achieved a CR of 100% vs 85% CR in the standard (daunorubicin 60 mg/m^2^ dose) group (p= NS). In the dose-intensified GO-based induction group, there were no cases of veno-occlusive disease noted. Additionally, side effects, such as cytopenias, febrile neutropenia, pneumonia, and cardiac toxicities, were comparable in both the intensified and non-intensified arms [1].

The ALFA-701 study noted thrombocytopenia (< 50 k/mm^3^) on Day 45 after chemotherapy in 22 patients (16%) in the GO group and four (3%) patients in the control group (p <0.0001). Also, the median number of platelet transfusions was significantly higher in the GO group [4]. Thus, the risk of GO causing worsening thrombocytopenia is certainly concerning but the study by Singh A et al. did not reveal a statistically significant difference in time to platelet recovery between the higher (41.5 days) and lower doses (39.5 days) of the daunorubicin cohorts and used a higher threshold of 100 k/mm^3^ platelets to constitute recovery. Our patient’s platelet recovery was 28 days (>100 k/mm^3^) after induction. However, our patient had a profound nadir of platelet count with < 10 k/mm^3^ for 11 days. She had a similar nadir period after her first consolidation cycle requiring transfusions. She has been noted to have human leukocyte antigen (HLA) antibodies, which have contributed to the thrombocytopenia picture and her response to transfusions. Nonetheless, further attention should be paid to the toxicity of thrombocytopenia in the context of adding GO to higher doses of daunorubicin.

## Conclusions

In the real-world setting, when NGS results may not be immediately available and there is an urgency to treat, one would need to consider starting 90 mg/m² of daunorubicin and then adding GO in young patients with AML who have favorable risk features. Randomized controlled trials with a large sample size need to be done comparing the intensified vs standard doses of daunorubicin when combined with GO to determine the optimal dose and safety profile.
